# DNA Repair Mechanisms, Protein Interactions and Therapeutic Targeting of the MRN Complex

**DOI:** 10.3390/cancers14215278

**Published:** 2022-10-27

**Authors:** Claire McCarthy-Leo, Fatima Darwiche, Michael A. Tainsky

**Affiliations:** 1 Center for Molecular Medicine and Genetics, Wayne State University School of Medicine, Detroit, MI 48201, USA; 2 Department of Oncology, Wayne State University School of Medicine, Detroit, MI 48201, USA; 3 Molecular Therapeutics Program, Karmanos Cancer Institute at Wayne State University School of Medicine, Detroit, MI 48201, USA

**Keywords:** MRN complex, DNA repair pathway, variants of uncertain significance

## Abstract

**Simple Summary:**

The MRN Complex (MRE11-RAD50-NBS1) is responsible for initiating DNA double-strand break repair and activating several downstream proteins. These proteins have been studied for their individual roles in inherited cancers and are included on several genetic testing panels. However, large amounts of genetic data remain a challenge to interpret due to the presences of variants of uncertain significance (VUS) which prevent proper variant classification and hinders clinical decision making. In this article, we will discuss the current role the MRN complex plays in the DNA repair pathway, the variants present in all three MRN complex genes and the MRN complex as a target for anti-cancer treatment.

**Abstract:**

Repair of a DNA double-strand break relies upon a pathway of proteins to identify damage, regulate cell cycle checkpoints, and repair the damage. This process is initiated by a sensor protein complex, the MRN complex, comprised of three proteins-MRE11, RAD50, and NBS1. After a double-stranded break, the MRN complex recruits and activates ATM, in-turn activating other proteins such as BRCA1/2, ATR, CHEK1/2, PALB2 and RAD51. These proteins have been the focus of many studies for their individual roles in hereditary cancer syndromes and are included on several genetic testing panels. These panels have enabled us to acquire large amounts of genetic data, much of which remains a challenge to interpret due to the presence of variants of uncertain significance (VUS). While the primary aim of clinical testing is to accurately and confidently classify variants in order to inform medical management, the presence of VUSs has led to ambiguity in genetic counseling. Pathogenic variants within MRN complex genes have been implicated in breast, ovarian, prostate, colon cancers and gliomas; however, the hundreds of VUSs within *MRE11, RAD50,* and *NBS1* precludes the application of these data in genetic guidance of carriers. In this review, we discuss the MRN complex’s role in DNA double-strand break repair, its interactions with other cancer predisposing genes, the variants that can be found within the three MRN complex genes, and the MRN complex’s potential as an anti-cancer therapeutic target.

## 1. Introduction

Maintaining the integrity of DNA is vital for cellular function; however, exogenous factors (i.e., irradiation, UV light, chemical agents) and endogenous factors (i.e., reactive oxygen species, lipid peroxidation, endogenous estrogens and alkylating agents) often result in DNA damage [1,2,3,4,5]. These factors can cause single- (SSB) or double-strand breaks (DSB) to the DNA requiring repair before DNA replication can occur. Inability to repair DNA damage often leads to cell death and incorrect repair of the DNA, causing genetic changes such as point mutations, duplications, deletions, translocations and DNA fusions resulting in significant consequences such as the development of cancer [1,2,3,4,6].

**Figure 1:**
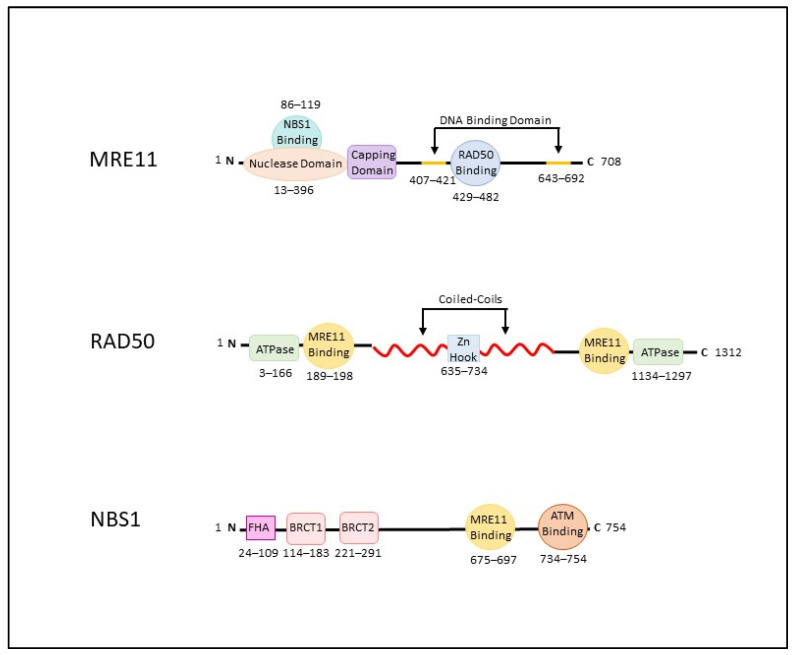
Key domains of the MRN complex. A schematic representation of the three genes that make up the MRE11, RAD50, and NBS1. MRE11 is 708 amino acids (aa) in length. RAD50 is 1312 aa and NBS1 is 754 aa. Domain location within each transcript in indicated (aa) below. (FHA: Fork-head associated domain; BRCT: BRCA1 C-terminal domains).

Cellular response to DNA damage includes the identification of damage, chromatin modification at the source, protein recruitment for the regulation of cell cycle checkpoints and repair of the DNA [7,8,9]. The MRN complex is a set of proteins that acts as a sensor in the initial stages of DSB repair to bind damaged DNA, tethers the broken ends for DNA repair, and recruits the ataxia-telangiectasia mutated (ATM) protein to the site of DNA damage, initiating the DNA repair pathway [7,8,9]. At the molecular level, the MRN complex consists of three proteins: the meiotic recombination 11 homolog 1 (MRE11), DNA repair protein RAD50, and phosphopeptide-binding Nijmegen breakage syndrome protein 1 (NBS1) (Figure 1). In addition to its ability to assist in DSB repair mechanisms, the MRN complex has also been reported to play an important role in telomere maintenance and meiotic recombination [7,8,9,10].

The MRN complex and many other DNA repair pathway proteins have been included on genetic testing panels used to identify hereditary cancer predisposition [11,12,13,14,15,16]. The increased use of multi-gene panel testing has led to a shift in genetic testing with challenges in sequence variant interpretation of the clinical significance based on current knowledge [11]. These variants of uncertain significance (VUSs) represent a challenge to accurate genetic counseling because clinicians cannot make medical management decisions based on VUS findings and misinterpretation has the potential to lead to inaccurate risk assessment of the patient and treatment planning [17,18,19]. Currently, there are numerous VUSs associated within *MRE11* (*n* = 701), *RAD50* (*n* = 1747), and *NBS1*, (*n* = 1377) [20]. In this review, we will discuss the role of the MRN complex proteins in DNA repair mechanisms and the impact mutations play on their functions, including other proteins with which they interact. Such proteins are often associated with cancer predisposition and are targets of current therapies. Additionally, we will review the current precision medicine approaches to exploit the MRN complex and its therapeutic potential.

## 2. The MRN Complex (MRE11-RAD50-NBS1)

The conformations and interactions of the proteins that make up the MRN complex enable both specific and diverse roles within the cell. The MRN complex has been reported to not only aid in the sensing and initiation of DNA DSB repair, but also to activate cell cycle checkpoints, as well as maintain telomeres and manage meiotic recombination [7,8,9,21,22,23,24,25,26]. A schematic of the three proteins that make up the MRN complex and important functional domains are further described in Figure 1.

*MRE11* (OMIM accession #600814) is located on chromosome 11 and is 708 amino acids (aa) in length. The core of the MRN complex consists of a MRE11 dimer that interacts with RAD50 via a specific domain (aa 429−82) and binds damaged DNA through 2 domains (aa 407−421; aa 643−692) [27,28,29,30]. In addition, each MRE11 molecule contains a nuclease domain and nuclease-capping domains (aa 1−342) [31]. The nuclease domain provides the MRN complex with the ability to cleave broken DNA ends through its 3′ to 5′ exonuclease, single-stranded DNA endonuclease activities and double-stranded DNA endonuclease activity that cleaves DNA-protein adducts to allow for repair [31,32,33,34,35]. The MRE11 nuclease domain is also the site of NBS1 binding (aa 86−119) [29]. The capping domain provides selectivity for DNA substrates via rotation resulting in the reorientation of the MRE11 dimerization axis [31]. This rotation has been suggested to control the ability to unwind double-stranded DNA by MRE11. Additionally, the MRE11 dimer acts as a surface for other DNA repair proteins and checkpoint factors [36].

The *RAD50* gene (OMIM accession #604040) encodes a 1312 aa protein that is located on chromosome 5. RAD50 is the largest subunit of the MRN complex, and is a member of the structural maintenance of chromosomes protein family [37]. The structure of an individual RAD50 polypeptide contains a long internal coiled-coil domain extending 500 aa in each direction that folds back upon itself bringing the N- and C-terminals together thus forming an ATPase head domain [36,38]. The apex at the other end of coiled-coil structure contains a central CxxC amino acid motif, which forms a zinc-binding motif, or zinc-hook, and mediates zinc-dependent interactions (aa 635−734) [28,39]. The RAD50 ATPase dimerizes in an Mg2+ and ATP-dependent manner [37]. The core RAD50 complex is arranged so that the DNA-binding sites on the MRE11 dimer (aa 189−198) are in proximity to the two RAD50 ATPase domains (aa 3−166; aa 1134−1297) [28]. The MRN complex binds to DNA causing the RAD50 coiled-coils to zip up and form a clamp around the DNA molecule, allowing for reorientation of the MRE11 dimer to the side of the complex [40]. The coiled-coils tether damaged DNA ends and acts as a gate for the recognition and organization of DNA molecules for repair [40]. RAD50 is a critical player in maintaining genomic stability and is required for cell-cycle checkpoint signaling through the MRN complex [41,42].

Located on chromosome 2 and encoding a protein 754 aa in length, *NBS1* (OMIM accession #602667) functions as a regulatory docking protein containing a single phosphopeptide-interacting forkhead associated (FHA) domain (aa 24−109) and two BRCA1 C-terminal (BRCT) domains (BRCT1 aa 114−183; BRCT2 aa 221−291) in its N-terminus [42,43]. On its C-terminus, NBS1 contains an MRE11 binding domain (aa 675−697) and an ATM-binding domain (aa 734−754) [28,42]. NBS1 contains a nuclear localization signal sequence for the MRN complex and its interactions are regulated by the binding of multiple phosphorylated proteins to these domains [42,44,45,46]. NBS1 interacts with the MRN complex through interactions with MRE11 [42]. This interaction stabilizes the complex, changes the substrate specificity for the endonuclease activity, alters activities of RAD50, enables the opening of fully paired DNA hairpins, and partially unwinds DNA [41] 

## 3. MRN Complex and Initiation of DNA Double-Strand Break Repair

**Figure 2:**
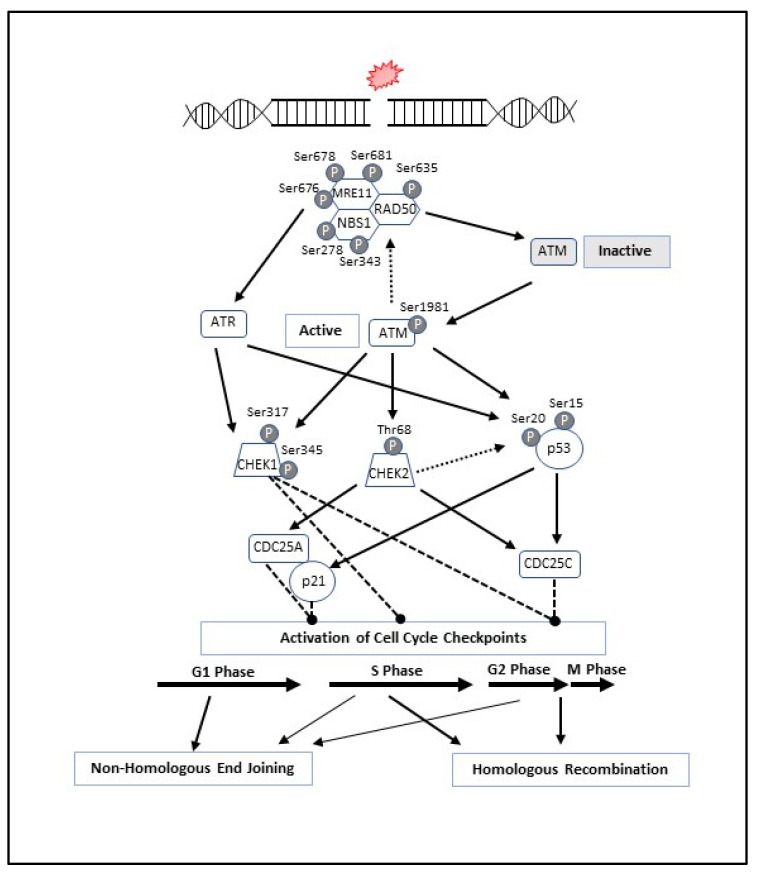
DNA Repair Pathways; Protein Recruitment and Cell Cycle Activation. After a DSB, The MRN complex locates to the site of damage and recruits and helps activate ATM stimulating its phosphorylation at Ser1981. Activated ATM phosphorylates all three MRN proteins; NBS1 on sites Ser278 and Ser343, RAD50 on site Ser635, and Mre11 on sites Ser676, Ser678 and Ser681. The activation of ATM results in multiple downstream events including activation of p53 by phosphorylation at Ser15 and Ser20, and CHEK2 at Th68. Active p53 and CHEK2 in-turn activate different cell cycle checkpoints. Activation of ATR results in the phosphorylation of CHEK1 on Ser317 and Ser345. ATR can also catalyze phosphorylation of p53 at Ser15 and Ser20 as the catalytic subunit of DNA-PKcs. The activation of cell cycle checkpoints at different stages often determines the method of repair and which additional proteins need to be recruited. In addition to the MRN complex, each method of repair replies upon other proteins involved in the pathway to facilitated DNA double-stranded repair.

When a DSB occurs, the MRN complex locates to the site of DNA damage where it recruits and activates Ataxia-Telangiectasia Mutated (ATM) through the direct interaction with NBS1 [47]. The activation of ATM initiates autophosphorylation at Ser1981 and the phosphorylation of all three MRN proteins, initiating downstream signaling [48,49]. Specifically, ATM phosphorylates NBS1 on two sites (Ser278 and Ser343), RAD50 at a single site (Ser635), and MRE11 on sites Ser676, Ser678 and Ser681 [48,49,50,51,52,53,54,55,56,57,58,59]. The ATM-dependent phosphorylation of the MRN complex results in a conformational change of the complex from an open to closed formation, thus activating the complex to bind to DNA [60]. Once activated, ATM phosphorylates downstream proteins including checkpoint kinase 2 (CHEK2), checkpoint kinase 1 (CHEK1), tumor protein p53 (TP53 or p53), H2AX, BRCA1, and CtIP (Figure 2) [48,61,62].

CHEK2 activation occurs via phosphorylation by ATM at Thr68, resulting in dimerization of CHEK2 through Thr68-phosphopeptide-binding FHA domain interaction, which leads to genomic stabilization at the site of DNA damage repair [63,64]. Active CHEK2 can arrest the cell cycle in G1/S or G2/M phase by phosphorylation of CDC25A or CDC25C, respectively. Additionally, arresting the cell cycle in G1/S phase can be promoted by CHEK2 via phosphorylation of p53. Once phosphorylated by ATM and/or CHEK2, active p53 induces G1 arrest through p21 activation. Additionally, p53 is involved in maintaining the G2/M checkpoint by transcriptional repression of CDC25C. 

ATM and Rad3-related (ATR) activation occurs in response to SSBs, resected DSBs, and deoxyribonucleotide triphosphate depletion [65,66]. The activation of ATR results in the phosphorylation of CHEK1 on Ser317 and Ser345 [54]. There are less data supporting the role of MRN-mediated ATR signaling in response to DNA damage, however, RAD50 is initially required for ATR activation and in-turn is phosphorylated by ATR. Phosphorylation of RAD50 in also required for cell cycle control and survival through CHEK1. CHEK1 activation by ATM and ATR is integral for intra-S and G2/M checkpoint responses before cell division [67,68].

**Figure 3:**
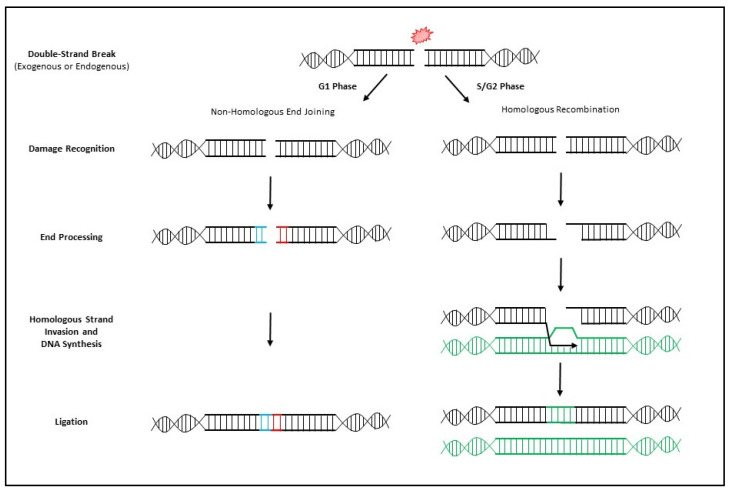
Basic Mechanisms of Classical Non-Homologous End Joining (NHEJ) (**Left**) and Homologous Recombination (HR) (**Right**). Classical NHEJ is predominantly initiated when a DNA double-strand break (DSB) occurs in G1. The MRN complex in cooperation with other proteins binds the damaged DNA, tethers the broken ends together for end processing, priming and ligation. This process is often error-prone and often results in the permanent loss of bases at the DSB site. A DSB in S or G2 most often initiates HR and relies on the presence of a homologous sister chromatid of DNA to prevent loss of genetic information. The DSB is identified by the MRN complex which initiates 5′ end resection. The resulting 3′ single-stranded overhang is bound by proteins and invades the homologous strand of DNA and initiates DNA synthesis. This results in no loss of genetic information.

The stage of the cell cycle in which a DSB occurs determines the exact mechanism by which DNA repair will be employed (Figure 2 and Figure 3). Figure 3 is an overview of the two primary mechanisms of DNA DSB repair. Non-homologous end joining (NHEJ) is a mechanism of DSB repair that is primarily used when the cell is in G1, requiring no homology with a second DNA duplex or between two damaged ends of DNA [69,70,71]. This mechanism relies upon the MRN complex binding to the damaged DNA and tethering the broken ends together for resection, priming and gap filling [27,40,71]. While NHEJ repairs DSBs, this process often results in the permanent loss of bases at the site of a break; therefore, NHEJ is considered an error-prone mechanism of repairing DSBs. Alternatively, homologous recombination (HR) is employed to repair DSBs in either S or G2 phase when sister chromatids are present [53,72]. HR requires a sister chromatid or homologous chromosome and because the repair mechanism relies on a homologous strand, it is considered a high-fidelity repair of genetic information.

BRCA1 is rapidly phosphorylated by ATM after DNA damage, which is involved in both HR and NHEJ [73,74,75]. BRCA1 plays a role in HR- mediated DNA damage repair though CtIP and is associated with the MRN complex interaction [27,29,36,76]. Phosphorylation of CtIP by ATM resulting in CtIP-BRCA1 binding, is required for the interaction of BRCA1 with NBS1 [76,77]. Additionally, CtIP and DNA-dependent protein kinase (DNA-PK) are required for the endonuclease activity and DNA end processing of the MRN complex [45,78,79,80]. The BRCA1-CtIP-MRN complex is important for facilitating resection at the site of DSB and generation of a 3′ single-stranded overhang needed for HR-mediated repair [36,76]. This 3′ overhang invades the homologous strand of DNA and uses it as a template to repair the chromosome with the DSB. In NHEJ, ATM phosphorylates 53BP1, which acts as a scaffold protein and recruits RAP1-interacting factor 1 (RIF1) and Pax transactivation domain-interacting protein (PTIP), which are pro-NHEJ proteins, to the DSB thereby inhibiting HR [81,82,83]. 53BP1 inhibits BRCA1 recruitment to the site of a DSB in G1 and inversely BRCA1-CtIP inhibits 53BP1 and RIF1 recruitment to the site of a DSB in S/G2 phase [83]. This association suggests an accessory role of BRCA1 in NHEJ. 

Downstream in the HR-mediated DNA damage pathway, BRCA1 recruits BRCA2 and RAD51 through a coiled-coil interaction with PALB2 [84,85]. Once phosphorylated by ATM, PALB2 functions as partner to BRCA2 and co-localizes to the site of DNA damage where RAD51 is subsequently loaded onto resected 3′ single-strand overhangs after nucleotide resection [84,86,87,88,89,90]. The loading of RAD51 onto replication protein A (RPA)-coated single-strand DNA (ssDNA) is facilitated by BRCA2 [91]. In addition to its co-localization with PALB2, the BRCA2 protein can also prevent the dissociation of RAD51 from the ssDNA. Once loaded onto the ssDNA via BRCA2 and PALB2, RAD51 is involved in the search for homology and strand pairing following the invasion into a homologous strand of DNA [92].

## 4. Mutations within the MRN Complex and Human Disease

Given the important role the MRN complex plays in initiating DSB repair, it is anticipated that inherited deficiencies would result in the development of disease. Rare autosomal mutations within *MRE11, RAD50* and *NBS1* can result in genomic instability disorders [93,94,95,96,97,98,99]. A-T is an autosomal recessive genetic disorder, which results in a broad range of phenotypes affecting the nervous and immune systems [100,101,102]. While the classic presentation of A-T is due to mutations in *ATM*, mutations within *MRE11* phenotypically present in a similar manner resulting in the classification A-T-like disease (ATLD; OMIM accession #604391) [93,103,104]. ATLD is a genetic disorder caused by biallelic pathogenic mutations of MRE11 and clinically presents with progressive cerebellar degeneration and ataxia [93,103,104]. ATLD-diagnosed patients with mutant *MRE11* often contain mutations located within MRE11′s highly conserved N-terminus, which contains its nuclease domain and the site of NBS1 binding, altering protein function [105]. 

Defective NBS1 and RAD50 proteins have also been linked to disease. Nijmegen breakage syndrome (NBS; OMIM accession #251260) is a genetic disorder with 90% of cases being caused by the Slavic founder mutation, 657del5 (rs587776650) within the *NBS1* gene [94,95]. Given the NBS1 role in DNA repair and telomere maintenance, NBS is characterized by chromosomal instability resulting in complex neurological abnormalities [27,96,98]. Similarly, mutations within *RAD50* result in a genetic disorder that clinically presents with neurological developmental defects, resulting in the designation as NBS-like disorder (NBSLD; OMIM accession #613078) [27,98]. *RAD50* mutations that result in NBSLD are either homozygous or compound heterozygotes [97,98,99]. For example, one patient contained compound heterozygous *RAD50* mutations, with a single nonsense mutation 3277C > T (p.R1093 *) on one allele, and a point mutation 3939A > T (p.Ter1313Tyr) on the other allele resulting in an altered termination codon that extended the reading frame by 66 aa on the other allele [97,98]. Additionally, a splicing defect was observed in another patient which resulted in a homozygous point mutation c.2524G > A (p.Val842Ile) in *RAD50* [99].

In addition to ATLD, NBS and NBSLD, heterozygous germline mutations within the MRN complex have been shown to predispose individuals to various cancers. *MRE11, RAD50* and *NBS1* have been classified as moderately penetrant genes for breast cancer [28,106,107]. More specifically, NBS1 demonstrates epidemiological evidence of a strong association between variants and breast cancer risk [108,109]. As the MRN complex has been shown to be involved in telomere maintenance, BRCA1 has also been implicated in the regulation of telomere length and stability [110]. RAD50 has been shown to recruit BRCA1 to the telomeres and BRCA1 inhibits telomerase activity while simultaneously maintaining telomere stability via the telomere capping domain [110]. There is also evidence to suggest that telomere maintenance function of the MRN complex drives senescence after DNA damage [111,112]. In addition to breast cancer, variants within the MRN complex genes have been associated with the development of ovarian, prostate, colon cancer and gliomas [28,107,113,114,115,116,117,118,119,120]. 

## 5. MRN Complex Interaction with Cancer-Predisposing Genes

When mutations occur within proteins in DNA DSB repair pathway, the ability of the cell to find and repair damaged DNA and activate cell cycle checkpoints, can be compromised. Downstream signaling from the MRN complex initiates the activation of cell cycle arrest and DNA repair through ATM, CHEK2, ATR, CHEK1, p53, BRCA1, BRCA2, PALB2, and RAD51, all of which have been shown to contain mutations linked to the predisposition of malignancy [37,38,39,41,42,43,76,77,108,121,122,123,124,125,126]. 

Mutations within *ATM* that disrupt the protein’s ability to phosphorylate the MRN complex can inhibit the cells capability to initiate the DNA repair pathway. Heterozygous germline or somatic mutations in *ATM* have been associated with the development of breast, ovarian, bladder, and melanoma cancers [76,108,124,126]. Mutations within *ATM* and their individual roles in disease development are discussed further in reviews by Choi et al., Amirifar et al., and Levy et al. [73,74,75].

Once activated, CHEK2 plays a key role in DNA damage response by acting on multiple substrates involved in the cell cycle, apoptosis, and gene expression. As a key player in cellular response to DNA damage, germline and somatic mutations within *CHEK2* have been linked to the development of ovarian, breast and prostate cancer [77,108,121,124].

As another important avenue of DNA damage response involved in cell cycle control, mutations in ATR and CHEK1 also have roles in the development of certain cancers. More specifically, *CHEK1* germline and somatic mutations have been observed in cancers such as breast, endometrial, colorectal and stomach, and germline mutations within *ATR* have been linked to an increased risk of developing oropharyngeal cancer and other malignancies [127,128,129,130,131,132,133,134].

The p53 tumor suppressor is activated by phosphorylation in response to DNA damage through MRN-dependent activation of ATM and CHEK2. The p53 protein plays an important role in transcriptional activation of cell cycle arrest and apoptosis. Somatic mutations in *TP53* are the most commonly acquired mutations (>50%) in human cancer, while germline mutations in *TP53* are the major risk alleles in Li-Fraumeni Syndrome [86,135].

Inheritance of a *BRCA1* mutations has been associated with an increase in an individual’s risk of developing breast, ovarian, prostate, and pancreatic cancers [85,87,123,124,136]. Mutations within the MRN complex and *BRCA1* may alter the cell’s ability to cause cell cycle arrest and prevent senescence ultimately leading to cellular immortality and potentially cancer [110,111,112]. 

The lack of functional PALB2 results in impaired loading of BRCA2 and RAD51 to sites of DNA damage. Germline mutations within *PALB2* have been associated with breast, ovarian, pancreatic, fallopian tube and peritoneal carcinomas [84,88,89,109,122,124]. Germline mutations within *BRCA2* increase an individual’s risk of developing breast, ovarian and pancreatic cancers [123,124,136]. *RAD51* mutations have led to either over- or under- expression of the protein. Over-expression of RAD51 protein has been observed in many cancers including include breast, ovarian, pancreatic, head and neck squamous cell carcinoma (HNSCC), prostate, and non-small-cell lung cancer [56,57,90,124]. Under-expression of RAD51 has been observed only in renal cell carcinoma [91]. It is also interesting to note that in the treatment of germline BRCA1/2 breast cancer, expression of RAD51 nuclear foci can act as a biomarker for PARP inhibitor resistance [137]. 

## 6. Assessing Mutation Impact within the MRN Complex Using Functional Studies

**Figure 4:**
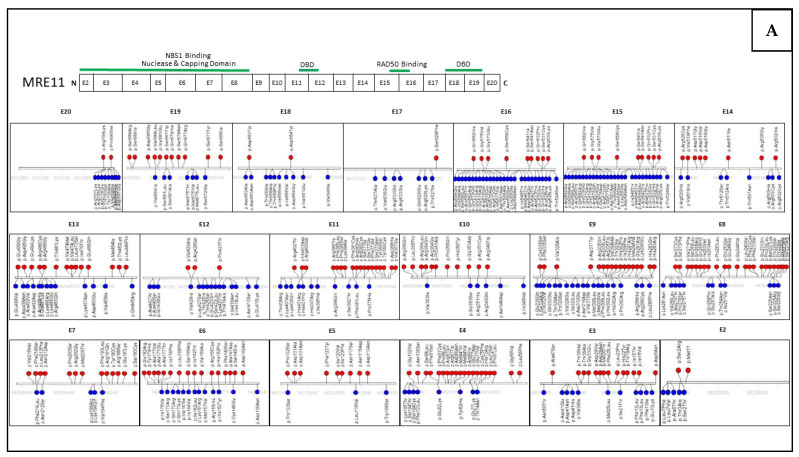

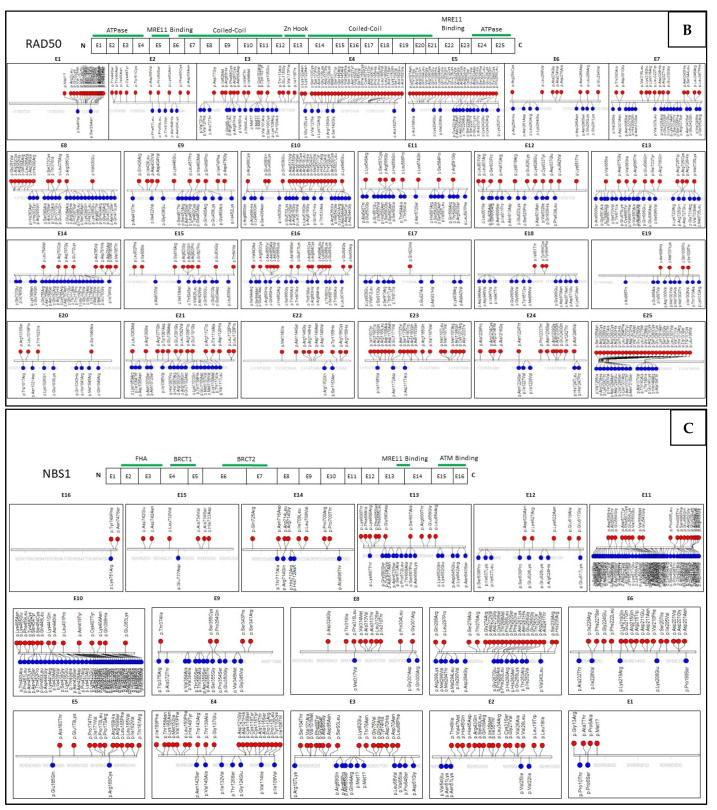
These data were collected from the Genome Aggregation Database (gnomAD), we identified 410, 659 and 381 missense mutations within the exons of *MRE11* (**A**), *RAD50* (**B**), and *NBS1* (**C**), respectively. SIFT predictive scores were collected for each mutation and are classified as deleterious (red) or tolerant (blue). The key domains of each gene are correlated to their location within the exomes.

The use of multigene panel testing for hereditary cancer predisposition syndromes has increased the identification of mutations in many genes including those that encode the MRN complex [138]. Several of these mutations in the MRN complex have been associated with disease and functionally evaluated for why these mutations result in disease. However, there is a lack in relevant clinical data to properly classify such variants as either pathogenic (cancer-causing) or benign, which has led to ambiguity in genetic counseling. In silico bioinformatics tools have been developed to predict the pathogenicity of missense variants based on evolutionary conservation of an amino acid of interest [139]. However, these tools can only be used as supporting criteria for classifying variants according to the guidelines of the American College of Medical Genetics and Genomics and the Association for Molecular Pathology [18]. The in silico bioinformatics tool, SIFT (Sorting Intolerant From Tolerant) is one of these tools that predicts the functional impact that a single nucleotide polymorphism has on protein function [92]. In comparison to several in silico tools, SIFT has the highest accuracy and sensitivity when predicting missense variants in a single gene [140,141]. Figure 4 contains the SIFT predictions using of all missense variants in MRE11, RAD50 and NBS1 collected from the Genome Aggregation Database (gnomAD) large-scale sequencing database. The presence of hundreds of SIFT-predicted pathogenic mutations within MRE11, RAD50, and NBS1 and the inability to use this data to make clinical decisions points to the need for further investigation into the functional effects of these mutations. Well established in vivo and in vitro functional analyses, in combination with the supporting data from in silico bioinformatic tools, can be applied to determine whether a mutation has an impact on patient risk of developing cancer.

In *MRE11*, sequences with a higher presence of SIFT-classified deleterious mutations are in the exons that contain the capping, nuclease and NBS1 binding domain. (Figure 4A). These domains are highly conserved and important to the MRN complex function and DSB repair, so mutations within this region would have a greater impact on protein function. It is interesting to note that a high number of mutations classified as tolerant by SIFT occur within the RAD50 binding site and DNA binding domains. This may be due to similarity in amino acid properties, or low impact to the overall function of the domains. In RAD50, a greater number of deleterious-predicted mutations impact the ATPase domains as well as the MRE11 binding sites, while tolerant-predicted mutations in the coiled-coil domains have minimal impact (Figure 4B). In NBS1, the FHA, BRCT1 and BRCT2 domains frequently contains amino acid variants, many of which are classified as deleterious by SIFT (Figure 4C). Many tolerant mutations can be found in exons 9−13 of *NBS1* which are consistent with regions that appear not to contain functional domains in all three genes. 

However, there are sites of predicted deleterious mutations within each of the three genes not located near any known functional domains. Mutations within these regions may have a greater impact on protein folding, thereby reducing the protein’s function. Although these algorithms are helpful in predicting the functional impact of individual mutations, a caveat is that these predictions may lack accuracy and are rarely employed in clinical genetic counseling. Many of these mutations will still need to be studied in experiments that comprehensively evaluate their functional impact on multiple protein targets. Functional studies can be tailored to any gene of interest to assess the impact mutations have on protein function. Furthermore, clinical guidelines could thereby take in account many pieces of evidence (predictive algorithms, functional studies, mutation location, etc.) to interpret and classify a sequence variant [18].

## 7. MRN Targeted Therapies and Synthetic Lethality in Cancer Treatment

As research continues to associate mutations within the MRN complex genes with the risk of cancer, the potential to develop targeted therapies is compelling. At present, the MRN complex is being studied in vitro and in vivo in combination with currently used chemotherapy agents for the treatment of breast, prostate, head and neck cancers as well as malignant gliomas [70,114,133,142,143,144,145,146,147,148,149,150]. The endo- or exo-nuclease activity of MRE11 has been a target with the development of the drugs such as Mirin, PFM01, PFM03, and PFM39. In mammalian cell lines in vitro, Mirin has been shown to inhibit MRN-mediated ATM activation and ATM-dependent phosphorylation of NBS1 and CHEK2 as well as the 3′ to 5′ exonuclease activity associated with MRE11 [70,146]. Additionally, the Mirin derivative PFM39, primarily inhibits MRE11 exonuclease activity in vitro whereas PFM01 and PFM03 block MRE11′s endonuclease activity [70]. Mirin has been tested in human malignant glioblastoma cell lines and shown increased sensitization and tumor cell death when used in combination with the alkylating agent, lomustine [147]. Additionally, Mirin demonstrated the ability to inhibit cell growth in a prostate cancer study analyzing whole genome sequencing and RNA-seq data of three prostate cancer cell lines and in MYCN-amplified neuroblastoma in a gene expression data set [133,148]. RAD50 inhibition via RNA interference in combination with the DNA-damaging drug cisplatin demonstrated a significant enhancement of the cytotoxic effects when tested in breast cancer cells in vitro [149]. In that study, RAD50 inhibition was tested with combination therapy using cisplatin–doxorubicin or cisplatin–paclitaxel and both agents significantly suppressed cell growth compared to cisplatin alone [149]. RAD50 knockdown via an adenoviral vector was also tested in combination with cisplatin in squamous cell carcinoma cell lines derived from head and neck tumor resections and observed significant tumor cytotoxicity and regression in vitro and in xenograft mouse models [150]. NBS1 was also targeted in combination with cisplatin in HNSCC in vitro and in a nude mouse xenograft model. This demonstrated significant increased chemosensitivity when employing a mutated NBS1 adenoviral gene transfer that induced a dominant-negative inactivation of the MRN complex [142].

Recently, the phenomenon of synthetic lethality has been of interest in the context of cancer therapy. Two genes that interact in a pathway in a “synthetic lethal” manner may, when individually inactivated, have a minimal effect on cell survival. However, loss or inactivation of both genes together can result in cell death [151,152]. This method can be used to identify individual novel cancer drug targets when a gene mutated in the germline experiences loss of heterozygosity in tumors [153,154]. Exploiting synthetic lethal interactions in cancer cells can provide the means to genetically target cancer cells that have two inactive alleles, while sparing healthy cells.

The functions and interactions among proteins can be exploited to target tumor cells. After the impairment of a single gene, the cell can compensate via another gene in the pathway to continue cellular function and replication. Exploiting this mechanism has been the strategy for anti-cancer drugs that aim to induce synthetic lethality, which has been demonstrated with the MRN complex and other DNA repair proteins. In a clinical trial investigating the use of an ATP-competitive checkpoint kinase inhibitor in combination with a topoisomerase I inhibitor to treat disseminated small-cell cancers, one patient showed a complete response [155]. After performing whole-genome sequencing, the investigators identified loss of heterozygosity and a mutation in the highly conserved D-loop of RAD50. This mutation severely impacted activation of ATM and when treated with the checkpoint kinase inhibitor and DNA-damaging chemotherapy it resulted in synthetic lethality. Another drug that has been developed to induce synthetic lethality in cancer cells, are protein inhibitors of the poly (ADP-ribose) polymerase (PARP) family [156]. PARP inhibition (PARPi) targets tumors that have deficient HR repair, such as homozygous BRCA1 or BRCA2 mutations, and causes additional deficiency to repair dsDNA breaks resulting in cell death [157]. PARPi has become a successful clinical cancer treatment as a single-agent and in combination with chemotherapy [158,159].

Although drug development investigations targeting the MRN complex genes are limited, these studies suggest a potential role for MRN-targeted therapies to improve response to current chemotherapeutic agents even in BRCA1/2 intact cells. One such study by Lajud et al. [143], investigated how downregulation of the MRN complex may in turn sensitize BRCA-proficient cells to PARPi treatment resulting in synthetic lethality in HNSCC. This study demonstrated that dual disruption of the DNA repair pathway by targeting the MRN complex significantly augmented sensitivity to PARPi in vitro and in a mouse model with HNSCC xenografts in BRCA1/2 proficient cells. In another study focused on the loss of MRE11 expression in endometrial cancer (EC), 521 EC tumors samples and 10 cancer cell lines were analyzed with 30.7% having a loss of MRE11 protein expression, significantly correlating to a loss of the other MRN-complex proteins [144]. The loss of MRE11 in the EC tumors demonstrated high sensitivity to PARPi suggesting that MRE11 could function a possible predictive biomarker or as a potential drug target for successful PARPi treatment in endometrial cancers. The MRN complex expression was also studied on tissue collected from patients with BRCA1/2-deficient epithelial ovarian cancers that have undergone PARPi treatment [114]. Immunohistochemical analysis detected a lack of expression of one or more of the MRN complex proteins in 41% of all samples, more prominently in low grade, type I EOC. This study demonstrated that an increase in sensitivity to PARPi occurred when MRE11 protein was knocked down. In another study, MRE11 inhibition was achieved using the drug Mirin in combination with a PARPi in BRCA2-defective cells and the drug combination resulted in significant cell death via apoptosis in vitro [145]. While many of these studies suggest a specific role for targeting MRE11 protein in augmenting the sensitivity to PARPi, there is a need for further investigation in all three MRN complex proteins.

**Figure 5:**
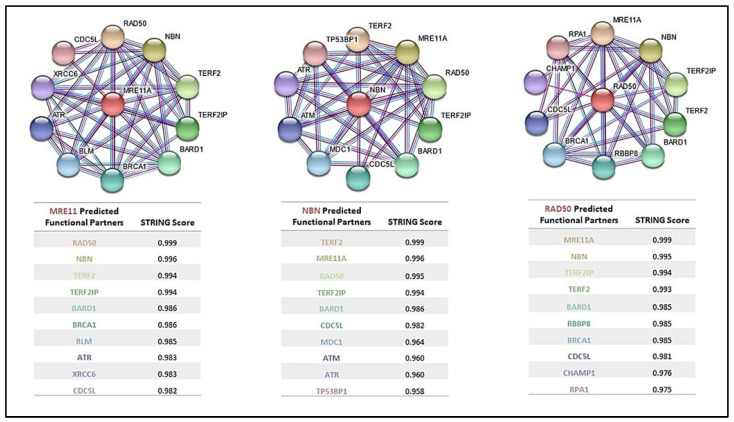
STRING Protein–Protein Interaction Networks of MRE11 (MRE11A), NBS1 (NBN), and RAD50. Each network and its subsequent table contain the top 10 predicted functional partners (PFP) of MRE11, NBS1, and RAD50 according to STRING (v11.0) https://string-db.org/ (accessed on 1 June 2022). Interacting Lines represent know interactions from curated databases (blue), experimentally determined (pink), or by co-expression (black). Scores represent the level of confidence a likely interaction is true on a scale of 0 to 1, with 1 being the highest possible confidence.

Protein–protein interactions that have been observed with the MRN complex proteins are depicted in Figure 5. The top 10 interactions using STRING (v11.0) have been scored using interaction data from curated databases, experiments, and co-expression [160]. Proteins that interact with all three MRN complex proteins include telomeric repeat-binding factor 2 (TERF2), TERF2-interacting protein 1 (TERF2IP), BRCA1-associated RING domain protein 1 (BARD1) and cell division cycle 5-like protein (CDC5L). These proteins are involved in telomere maintenance and protection, transcription regulation, and cell cycle control in response to DNA damage and have been also studied for their individual role in cancer development [116,117,118,119,120,138,139,151,152,153,154,160,161,162]. Additional proteins that interact individually with each of the MRN complex proteins are also indicated in Figure 5.

Because of the highly interactive role the MRN complex proteins play in DNA repair and the preliminary data supporting their role in cancer development, the MRN complex may prove an ideal target for synthetic lethality therapies. Additionally, the MRN complex proteins and those that interact with them, such as TERF2, TERF2IP, BARD1, CDC5L and others (Figure 5), could be a focus of directed combination targeted chemotherapeutics. All these proteins that have proven interactions are open opportunities for research of druggable targets exploiting synthetic lethality. This can be accomplished by performing a synthetic lethality screen of each individual gene via gene editing tools such as RNA interference or CRISPR-based technologies to systematically knock out genes and identify vulnerable gene pairs [163,164]. Furthermore, these gene pairs can be confirmed with drug targeting studies potentially leading to the development of novel anti-cancer therapeutics.

## 8. Conclusions

Because of its important role in the double-strand DNA repair pathway, the function of the MRN complex in disease development has been widely explored. While protein interactions with the MRN complex have been studied for their role in cancer development, there are many questions that remain regarding the complex. For example, next-generation sequencing has allowed for the identification of hundreds of single missense mutations within MRN complex but the field lacks the additional data regarding functional impact of these genetic lesions. Algorithms like SIFT can provide predictions to functional impact but lack a consistently accurate assessment to the mutational burden in vivo and are therefore not relied upon clinically. These genetics lesions pose a quandary as how to classify the effects of variations on protein function. With this lack of information, the genes that make up the MRN complex are currently considered to be moderately penetrant genes but not critical for the assessment of cancer development. VUSs within the MRN complex proteins demonstrate a gap in available information for risk assessment and clinical management.

Future studies should include how these individual proteins’ mutations affect the function of the whole MRN complex. The protein–protein interactions within the MRN complex and functional interactions with other proteins in the DNA repair pathway could also represent an area of polygenic risk assessment. Polygenic risk scores have been applied to genes that may contain multiple single nucleotide polymorphisms, which confer small risk individually, but their combined effect may be substantial. The combinatorial effect of mutations within the MRN complex and with other known cancer predisposing genes could be assessed and applied to the clinic for improved genetic surveillance and patient care through synthetic lethal targeting.

Improvements to genetic surveillance would also require addressing the identification and characterization of VUSs. Without accurate, evidence-based interpretation of these variants, genetic counselors are limited in their ability to provide medical management. Too often, patients are unable to receive definitive guidance regarding their genetic data and cannot take advantage of therapeutic measures that are available to carriers of known pathogenic mutations. In addition to identifying the relative pathogenicity of each individual variant, the development of comprehensive database containing functional implications of all single nucleotide variants in open reading frames could reduce the number of patients that receive the indefinite result of a VUS. Having all the up-to-date information in a centralized location, accessible to genetic counselors, would allow for the most detailed and reliable data to be applied for personalized patient care.
